# A Current Perspective on the Historical Geographic Distribution of the Endangered Muriquis (*Brachyteles* spp.): Implications for Conservation

**DOI:** 10.1371/journal.pone.0150906

**Published:** 2016-03-04

**Authors:** Bianca Ingberman, Roberto Fusco-Costa, Emygdio Leite de Araujo Monteiro-Filho

**Affiliations:** 1 Programa de Pós-Graduação em Ecologia e Conservação, Universidade Federal do Paraná (UFPR), Curitiba, Paraná, Brazil; 2 Wildlife Research Department, Instituto de Pesquisas Cananéia (IPeC), Cananéia, São Paulo, Brazil; 3 Zoology Department, UFPR, Curitiba, Paraná, Brazil; Centre for Cellular and Molecular Biology, INDIA

## Abstract

The muriqui (*Brachyteles* spp.), endemic to the Atlantic Forest of Brazil, is the largest primate in South America and is endangered, mainly due to habitat loss. Its distribution limits are still uncertain and need to be resolved in order to determine their true conservation status. Species distribution modeling (SDM) has been used to estimate potential species distributions, even when information is incomplete. Here, we developed an environmental suitability model for the two endangered species of muriqui (*Brachyteles hypoxanthus* and *B*. *arachnoides*) using Maxent software. Due to historical absence of muriquis, areas with predicted high habitat suitability yet historically never occupied, were excluded from the predicted historical distribution. Combining that information with the model, it is evident that rivers are potential dispersal barriers for the muriquis. Moreover, although the two species are environmentally separated in a large part of its distribution, there is a potential contact zone where the species apparently do not overlap. This separation might be due to either a physical (i.e., Serra da Mantiqueira mountains) or a biotic barrier (the species exclude one another). Therefore, in addition to environmental characteristics, physical and biotic barriers potentially shaped the limits of the muriqui historical range. Based on these considerations, we proposed the adjustment of their historical distributional limits. Currently only 7.6% of the predicted historical distribution of *B*. *hypoxanthus* and 12.9% of *B*. *arachnoides* remains forested and able to sustain viable muriqui populations. In addition to measurement of habitat loss we also identified areas for conservation concern where new muriqui populations might be found.

## Introduction

The muriqui (*Brachyteles* Spix, 1823) is the largest New World primate and is endemic to the Atlantic Forest of Brazil [1,2], which itself is considered one of the most endangered biomes in the world [3], with only 11.7% remaining, scattered in numerous fragments of varying sizes [4]. The two currently recognized species are *Brachyteles hypoxanthus* (Kuhl, 1820), the northern muriqui, and *B*. *arachnoides* (É. Geoffroy, 1806), the southern muriqui [5–7]. Both species are endangered due to habitat loss and poaching; *B*. *hypoxanthus* is considered critically endangered and *B*. *arachnoides* is endangered in the IUCN classification [8,9].

Evaluation of the conservation status of both was first based on habitat loss, as the historical geographic distribution was described in 1971[1], when the genus *Brachyteles* was considered monotypic. The distribution was later expanded to include new occurrence records [10–12] and split because of the reclassification into two species [7]. A more accurate definition of its distribution limits is still lacking, especially where the ranges of the two species are adjacent [8,13]. Uncertainties in the historical distributions for both species have important consequences for assessing their current conservation status. These uncertainties must be resolved to determine how much habitat is lost and where the muriqui may still potentially be found.

Knowing the original and current distribution records of a species is crucial for determining conservation status, which is often simply based on the restricted or reduced distribution of a species even when studies with detailed information are lacking [14,15]. Recently, interest in Species Distribution Modeling (SDM), based on the Hutchinsonian niche concept [16], has increased due to software development and access to species occurrence data [16–19]. Various mathematical algorithms have been developed (e. g. GAM, GLM, Maxent, Random Forest, GARP, SVM, ENFA) with the same objective: to identify areas suitable for the continued survival of species based on environmental variables [16,19]. These methods have been shown to be valuable in predicting possible habitat, even with presence-only data and a limited number of locality records [16,20–28] and are useful tools for determining distribution of species with little available information [21,27,29–32]. For several reasons (geographic barriers, biotic interactions, adaptation and anthropogenic changes to environments), few species are likely to occupy all suitable areas [17,22,29,33]. Thus, when research is focused on the area in which the species is known to occur, additional information and ecology-based hypotheses or assumptions should be added to the analysis to improve the estimates of both historical and current geographic distributions [17].

The recent development of SDM now provides us with new perspectives on species distribution limits and environmental factors that may influence these limits. Herein, we used SDM to model the potential distributions of *B*. *hypoxanthus* and *B*. *arachnoides*, and estimate their historical and current distributions. Based on these models, we discuss the geographic distribution of these species and how their conservation status may be better understood in the context of current fragmentation of their Atlantic Forest habitat.

## Materials and Methods

### Location data

We compiled locality data for *B*. *hypoxanthus* and *B*. *arachnoides* from the literature and from field and museum specimens, as available in the project “specieslink” (http://splink.cria.org.br) and those compiled by Aguirre [1]. When record coordinates were not available, we roughly estimated the coordinates by the use of figures and geographic information in the source publication. We used Google earth imagery to double-check location coordinates when necessary. Data collection and species observations tend to be grouped (sampling bias) due to the difficulty of accessing some locations [18,24]. Spatial clumping often results in spatial autocorrelation, which is an important source of bias in SDM, and often produces overestimation of the effective sample size, thereby inflating statistical significance and the probability of errors of commission (false positives) [34–36]. There is no consistent and well-researched methodology to analyze spatial autocorrelation in presence-only data [35], however, correcting for sampling bias has been shown to be very effective for increasing model performance and reducing commission errors [37–39]. To resolve sampling bias, we restricted the modeling calibration area (see background area below) [39–41] and we used spatial filtering in our experimental design [38,42] by using only one locality record within every 115 km^2^, which is based on the area that can support a minimum viable muriqui population over the long term (1,000 years cf. [43]) [24,27,42]. Within these constraints, we included only the most reliable geographical coordinates measured in the field, from published studies and latter from museum collections.

### Variable selection

We selected, as predictive variables, altitude and 19 bioclimatic variables from WorldClim (http://www.worldclim.org), comprising: annual mean temperature, mean diurnal range, isothermality, temperature seasonality, maximum temperature of the warmest month, minimum temperature of the coldest month, annual temperature range, mean temperature of wettest quarter, mean temperature of driest quarter, mean temperature of warmest quarter, mean temperature of coldest quarter, annual precipitation, precipitation of wettest month, precipitation of driest month, precipitation seasonality, precipitation of wettest quarter, precipitation of driest quarter, precipitation of warmest quarter and precipitation of coldest quarter. Bioclimatic variables were derived from the interpolation of monthly values of temperature and precipitation observed from 1950 to 2000 [44]. All variables were converted to a raster database with a resolution of 2.5 arc-minutes (c. to 5 km^2^ pixels). Initially we compiled bioclimatic information and altitude associated with each occurrence location separately by species. We avoided autocorrelation by excluding one variable from each pair of variables with very strong correlation (R^2^≥ 0.8[45]) [46,47]. Preference was given to altitude and climate extremes (i.e., Max/Min Temperature of Warmest/Coldest Month rather than Mean Temperature of Warmest Quarter, cf.[48]) because they are the most biologically meaningful for the muriquis [1].

### Statistical model and validation

While occurrence locations of each species were few, the expected distribution of each species was well-represented. Of the modeling methods, the maximum entropy algorithm is recommended in this situation because presence-only data are used and provide deterministic inferences from incomplete information [22] and is robust with relatively small sample sizes [16,20,21,24,25,27]. Maxent (version 3.3.3k, http://www.cs.princeton.edu/~schapire/maxent/) was used to model the potential distribution of each species. The dataset was randomly divided into 80% for training and 20% for testing, and modeled over 10,000 points of a background comprising the shape of the Atlantic Forest (IBGE shape biomes, http://downloads.ibge.gov.br/downloads_geociencias.htm) [39,49] and part of the Cerrado ecotone in the state of São Paulo, southern Brazil. We used bootstrapping with 500 randomly selected subsets for which model averaging was used. We analyzed the receiver operating characteristic (ROC) curve to determine model performance [20,22,25]. The area under the ROC curve (AUC) provides a unique probability (0, no discrimination, to 1, perfect discrimination) that indicates the quality of the result [20].

As a result of logistic output, the program Maxent generates a continuous prediction probability (0 to 1) of environmental suitability for species presence. We used the minimum training presence threshold (the lowest predicted suitability value at points where the species are found) to generate potential species distribution maps for a more conservative estimate [18,24].

### Post-modeling and interpretation

We used the geographic information program ArcMap10 to insert the cut-off threshold in the model and create maps. The Maxent output was transformed into raster and maps were interpreted in the context of our hypotheses. Because none of the SDM methods, including Maxent, considers dispersal limitation, results should be interpreted as potential distributions [17]. To create the historical distribution maps for the species, we identified areas likely to be distribution barriers [17,29] due to their geological characteristics (e. g. rivers), biotic interactions or areas where the species are known to be absent. Finally, we created a current distribution map by combining the historical distribution map and a map of Atlantic Forest fragments [50]. The difference (~1%) between the total forest remnant cover (10.6%) [51] and the 11.7% of Ribeiro et al. [4] is likely due to rounding error because the Fundação SOS Mata Atlântica & INPE [51] include only fragments larger than 0.03 km^2^, while Ribeiro et al. [4] includes all fragments. This potential error did not affect the reliability of the results of our analysis because we used only fragments larger than 1 km^2^.

## Results and Discussion

**Fig 1:**
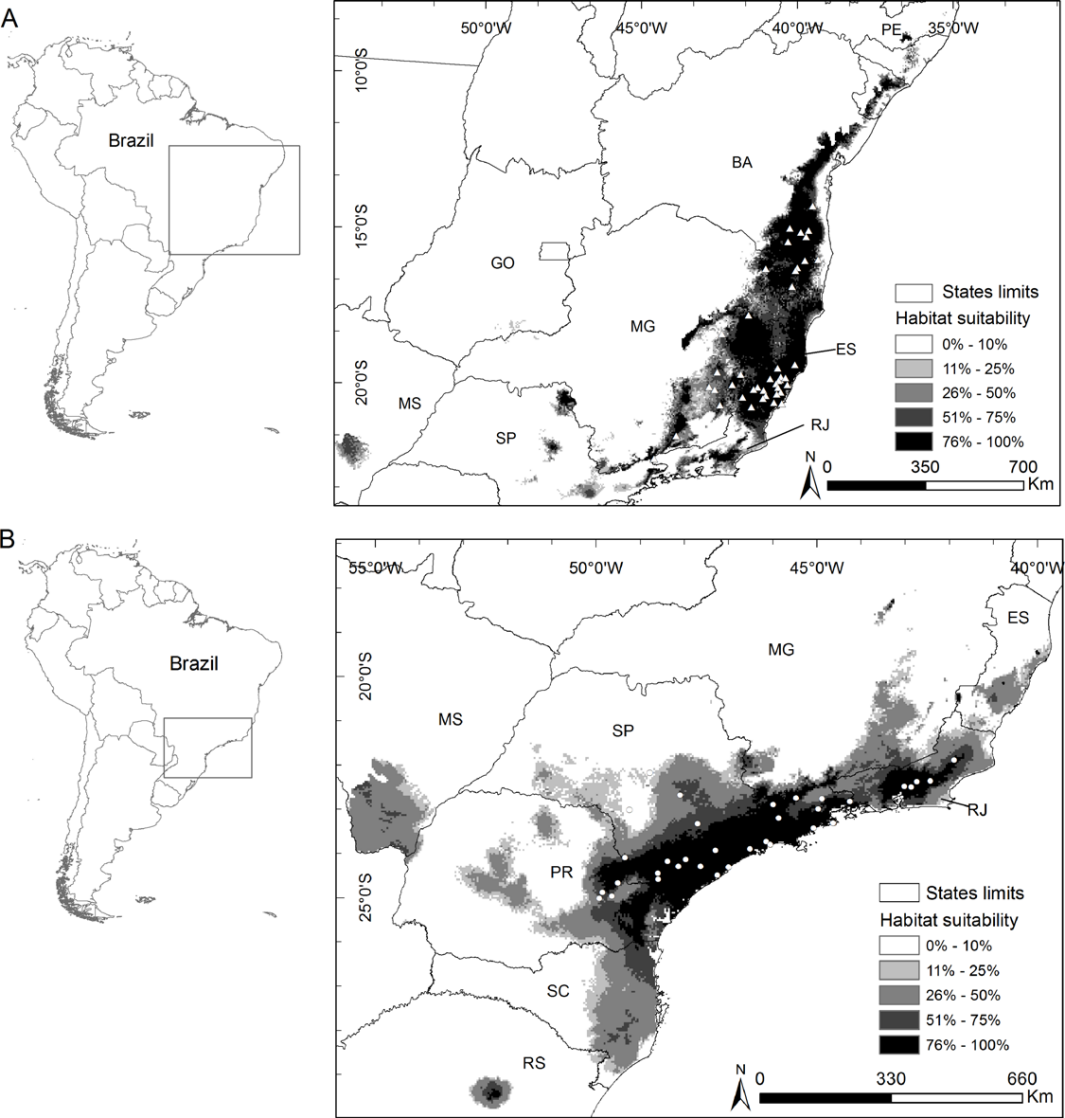
Environmental suitability map for the muriquis species. (A) *B*. *hypoxanthus* showing modeled presence localities as white triangles. (B) *B*. *arachnoides* showing modeled presence localities as white circles. Abbreviations of Brazilian states: PE–Pernambuco, BA–Bahia, GO–Goiás, MG–Minas Gerais, ES–Espírito Santo, RJ–Rio de Janeiro, SP–São Paulo, MS–Mato Grosso do Sul, PR–Paraná, SC–Santa Catarina and RS–Rio Grande do Sul.

We found 58 locations for *B*. *hypoxanthus* and 44 for *B*. *arachnoides*, and, after excluding grouped points to resolve sampling bias, used 43 independent locations for *B*. *hypoxanthus* (S1 Table) and 34 for *B*. *arachnoides* (S2 Table). The model included seven variables for *B*. *hypoxanthus* (S3 Table) and nine variables for *B*. *arachnoides* (S4 Table). Potential distribution maps (Fig 1) resulted in significant results (AUC = 0.952 for *B*. *hypoxanthus* and AUC = 0.946 for *B*. *arachnoides*) and the suitable habitat cut-off thresholds were 0.1 for both species.

### Potential distribution

According to SDM, suitable habitat for *B*. *hypoxanthus* is found in several Brazilian states, from southeastern Pernambuco (PE) to northeastern São Paulo (SP), for a total potential area of occurrence of 329,445 km^2^. Some suitable patches were identified in southern Mato Grosso do Sul (MS), the border of southeastern Goiás (GO) and southwestern Minas Gerais, and northern and central São Paulo (Fig 1A).

SDM results for *B*. *arachnoides* indicated suitable habitat from central Espírito Santo (ES) to eastern Santa Catarina (SC), for a total potential area of occurrence of 345,760 km^2^. Suitable habitat patches were also found in southern Mato Grosso do Sul (MS), central Paraná (PR) and northeastern Rio Grande do Sul (Fig 1B), but these are outside the historical range (see below).

Suitable areas for both species overlapped at central and southern Espírito Santo (ES), northern and central Rio de Janeiro (RJ), southeastern Minas Gerais (MG), northeastern São Paulo (SP) and at isolated patches in central São Paulo (SP) and southern Mato Grosso do Sul (MS; Fig 1).

### Historical distribution

When combining species locality data with SDM, we observed that historical distribution was shaped not only by environmental factors. Thus, in order to describe the historical distribution of *B*. *hypoxanthus* and *B*. *arachnoides*, we considered another two hypotheses that might have shaped their distribution limits: physical barriers (rivers and mountains), and biotic interactions (close related species may be mutually exclusive).

**Fig 2:**
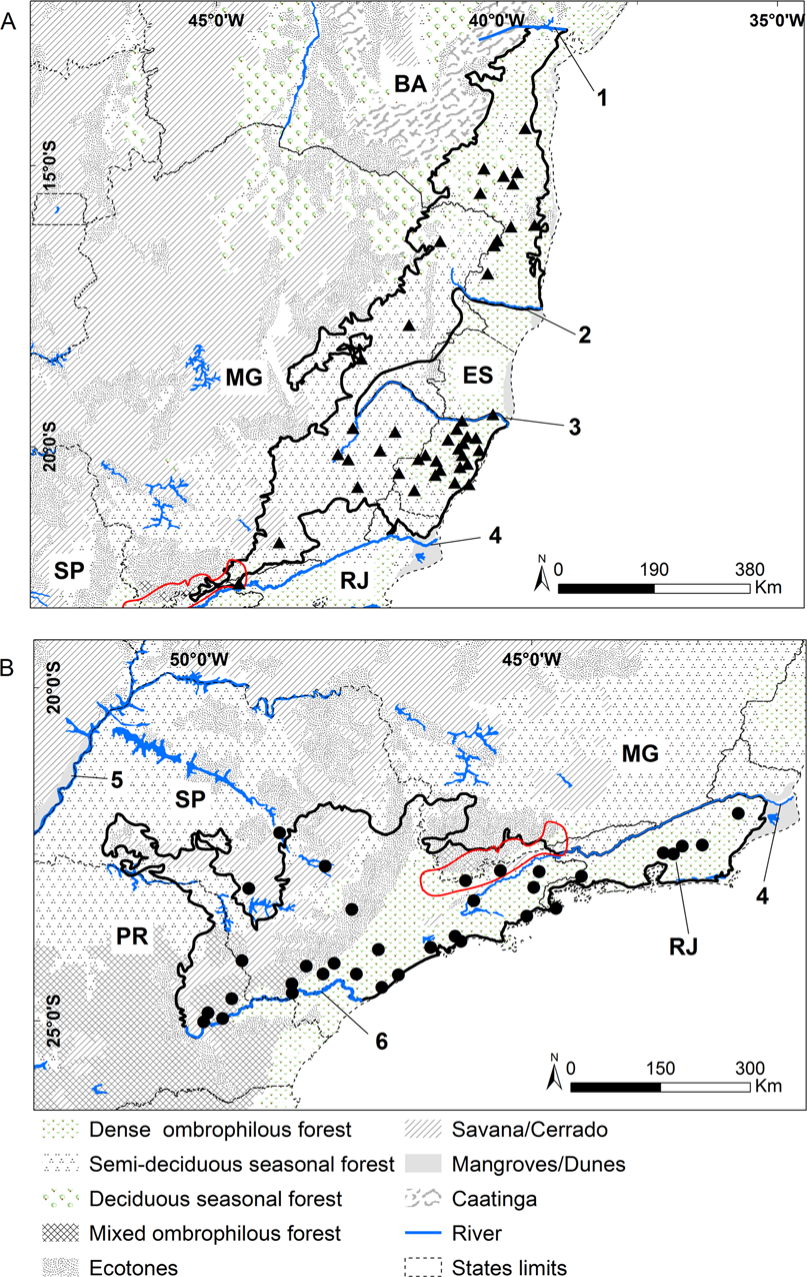
Historical distribution map for the muriquis species. (A) *B*. *hypoxanthus* showing modeled presence localities as black triangles. (B) *B*. *arachnoides* showing modeled presence localities as black circles. Historical distribution maps are indicated by a thick black line and the Serra da Mantiqueira region by a red line. Blue lines indicated rivers as stated below: 1 –Paraguaçu River; 2 –Itanhém (or Mucuri) River; 3 –Doce River; 4—Paraíba do Sul River; 5—Paraná River; 6 –Ribeira de Iguape River. State abbreviations, see Fig 1. * localities out of the historical range (georeferenced points refer to municipality, due to lack of the exact locality information).

The suitable habitat range for *B*. *hypoxanthus* may potentially extend north to Pernambuco (PE; Fig 1A). Since there is no current and historical occurrence records from central-eastern Bahia (BA), we assume that the northern limit of the historical range for *B*. *hypoxanthus* was the Paraguaçu River (BA) as suggested by Aguirre [1] (Fig 2A). The historical range follows environmental suitability comprising dense, deciduous and semideciduous forests and ecotones extending south to the Paraíba do Sul River at Rio de Janeiro state (RJ; Fig 2A). Even though the estimated suitable habitat range extends to central Rio de Janeiro (Fig 1A), occurrence records of the *B*. *hypoxanthus* are restricted to the northern margin of this river. The historical distribution of *B*. *hypoxanthus* reaches its southern limit at Serra da Mantiqueira region, a mountain range that crosses the states of Minas Gerais, Rio de Janeiro and São Paulo [52] (Fig 2A). We removed an unoccupied area between Itanhém River, southern Bahia, and Doce River, northern Espírito Santo *cf*. [1] from the potential distribution even though the entire region had (and still has) suitable habitat (Fig 2A). This distribution gap has also been observed for the maned sloth (*Bradypus torquatus*, Xenartha) [47]; for which, Moreira et al. [47] associated this pattern based on the vegetation type, as well as on the historical process of vegetation changes and retraction that occurred along the Quaternary. The vegetation hypothesis may also explain a similar gap for *B*. *hypoxanthus*; however, as there are also competing hypotheses (e.g. riverine barriers [53] and hunting associated with encroachment [1]), the reason why the muriqui is absent in this area remains unknown. After the post-modeling procedures, *B*. *hypoxanthus* historical area was estimated at 216,330 km^2^.

**Fig 3:**
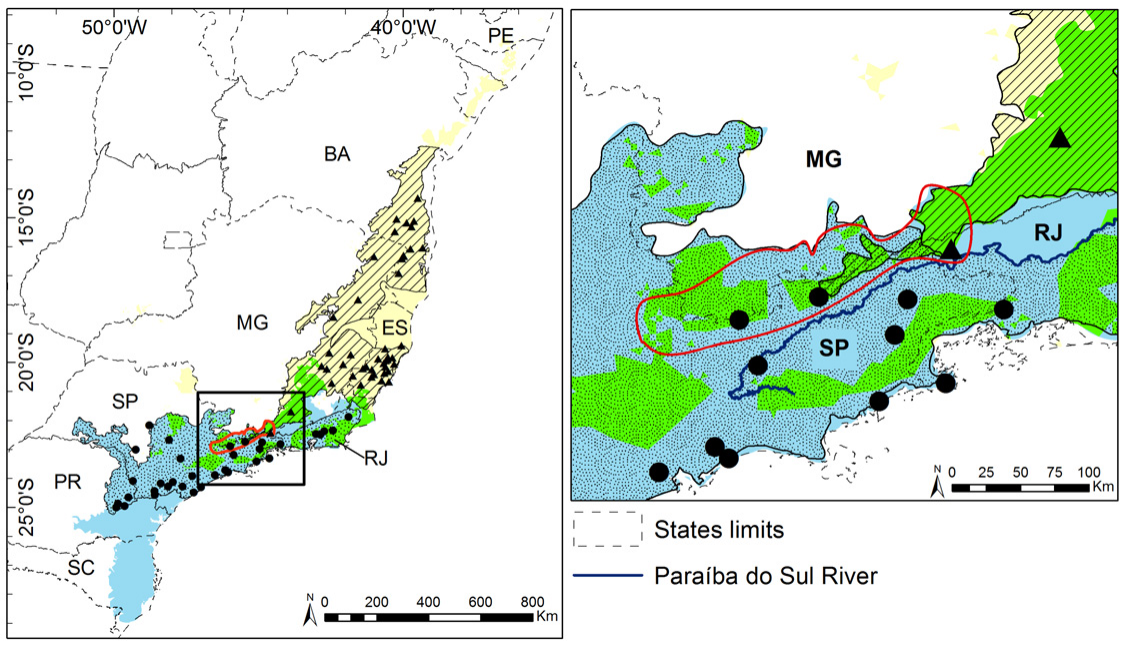
Map of the potencial distribution of muriquis species showing the range overlap of both species. *B*. *hypoxanthus* (black triangle) potential (yellow area) and historical (hatched area) distribution; *B*. *arachnoides* (black circles) potential (blue area) and historical (stippled area) distribution, showing range overlap (green area) of both species. Inset the Serra da Mantiqueira region (red line). State abbreviations, see Fig 1.

With respect to habitat suitability, the distribution of *B*. *arachnoides* may have reached the southeastern of Minas Gerais and central Espírito Santo (Fig 1B). However, observed *B*. *arachnoides* locations are restricted to the mountains of the Serra da Mantiqueira and the state of Rio de Janeiro, at the southern margin of the Paraíba do Sul River (Figs 2B and 3). Species Distribution Modeling (SDM) supports Aguirre's [1] hypothesis of occurrence in western São Paulo, although it does not seem to extend as far as the Paraná River. The historical range extends eastward through Paraná and gradually away from the border of São Paulo to the Ribeira de Iguape River (Fig 2B). Suitable habitat for the southern muriqui comprises mixed dense forest in central Paraná and the south following the coastal dense forest from southern São Paulo to Santa Catarina (Fig 1B). However, new evidence from field data indicates historical muriqui absence in areas south of the Ribeira de Iguape River, which supports the hypothesis that the river was a geographic barrier [54]. After the post modeling procedures, the historical area of occurrence was estimated at 159,880 km^2^.

Rivers seem to have limited dispersal of both muriqui species, delimiting their northern and southern historical boundaries. Riverine barriers shaped distribution limits for several species of primates [53,55,56] and it seems the muriquis was not an exception. Where the species are close together, the Paraiba do Sul River apparently limited both species ranges. However, this river limited the range for *B*. *arachnoides* only in Rio de Janeiro, since it is likely that this species had circumvented the head of the river in São Paulo, thus reaching the Serra da Matinqueira.

With the recognition of the two species of *Brachyteles* [6,7], it was first thought that only *B*. *arachnoides* was found throughout the Serra da Mantiqueira region, from São Paulo to Minas Gerais and Rio de Janeiro [57–59]. Once the *B*. *hypoxanthus* was found in Itatiaia National Park, in Rio de Janeiro (confirmed by more recent evidence [12,60]), both species are known to occur in Serra da Mantiqueira. However, there is still no evidence of co-occurrence, even within the contact zone predicted by our model (Fig 3). Herein, we propose two hypotheses to explain this apparent lack of spatial overlap: i) mountains with peaks up to 2,798 meters in the Serra da Mantiqueira region [61] acted as physical barriers; ii) the complete separation of these close related species are due to active avoidance of conflicting interactions [62–64], and consequently the establishment of one muriqui species has limited the spread of the other.

### Current distribution

The Atlantic Forest originally comprised 1,315,460 km^2^ [51] of which our study suggests that 376,210 km^2^ were inhabited by the genus *Brachyteles*. Because deforestation of the Atlantic Forest has not been uniform and only fragments greater than 1 km^2^ are considered suitable for potentially viable populations of muriqui (for 50 years [65]), we require more data to estimate true habitat loss for these species.

**Fig 4:**
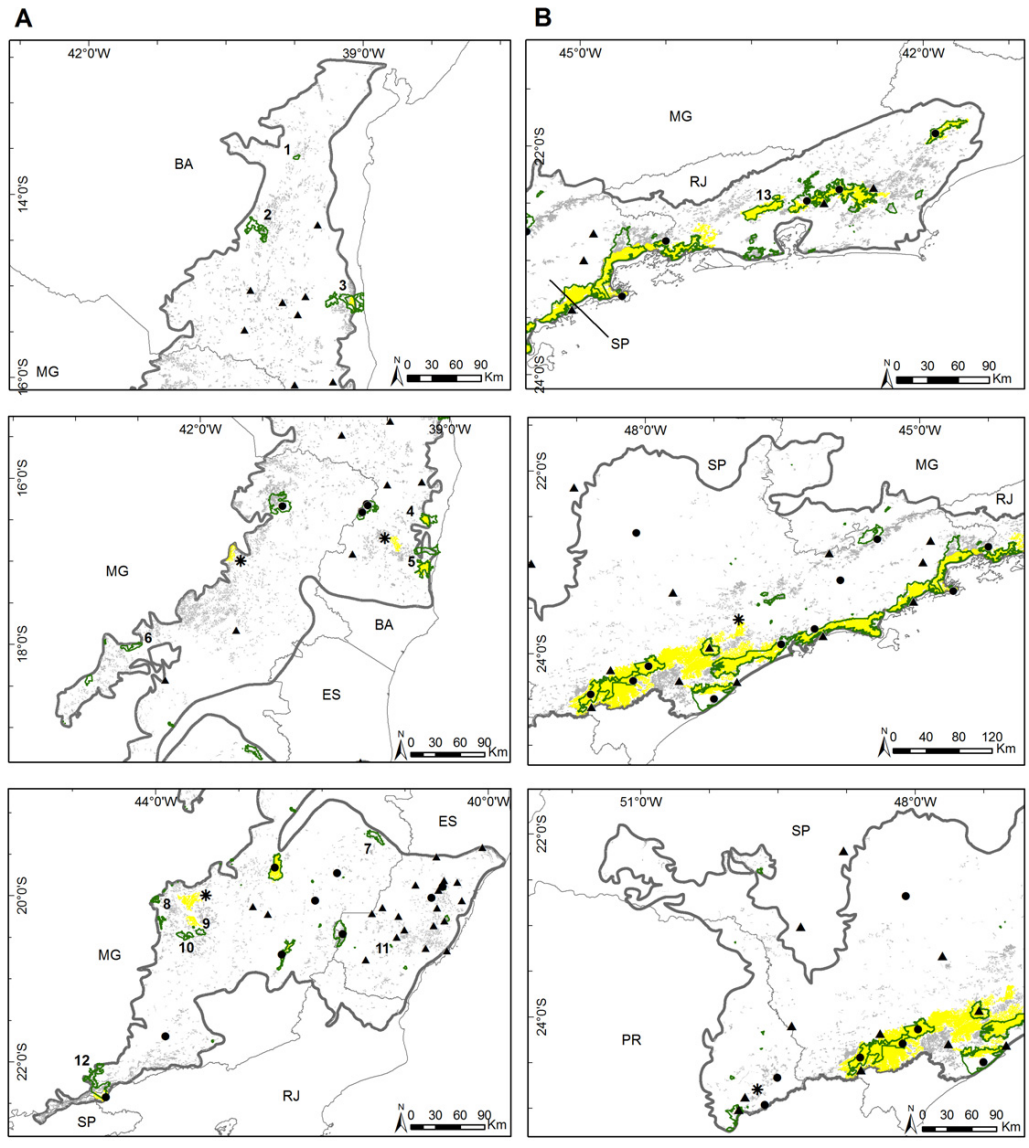
Historical distribution of muriquis species showing forest remnants and restricted protected areas. Forest remnants larger than 1 km^2^ (gray areas) and 115 km^2^ (yellow areas) highlighting the restricted protected areas (green line) inside the historical distribution (thick gray line) of (A) *B*. *hypoxanthus* and (B) *B*. *arachnoides*. Black circles represent current localities and black triangles represent historical localities. Restricted Protected areas: 1 –Wenceslau Guimarães Ecological Station; 2 –Boa Vista National Park; 3—Una Biological Reserve; 4—Pau Brasil National Park; 5 –Monte Pascoal National Park and Descobrimento National Park; 6 –Serra Negra State Park; 7 –Sete Salões State Park; 8 –Serra do Rola Moça State Park and Fechos Ecological Station; 9 –Itacolomi State Park; 10 –Serra do Ouro Branco State Park; 11 –Mata das Flores State Park; 12—Serra do Papagaio State Park; 13 –Tinguá Biological Reserve. * represents others indicated areas to survey. State abbreviations, see Fig 1.

Within the historical range of *B*. *hypoxanthus*, only 16,450 km^2^ (or 7.6%) of suitable forested habitat remains, scattered in numerous fragments larger than 1 km^2^. Several studies in forest remnants have slowly found new muriqui populations [12,52,66–75]. However, due to constant anthropic pressure, few of these remnants are still inhabited. In the most recent IUCN evaluation, there were only 12 known *B*. *hypoxanthus* populations in isolated forest fragments [8,73]. Today, 14 localities are known to have *B*. *hypoxanthus* [13], but SDM suggests that it might be found elsewhere, with approximately 14,580 km^2^ divided into 4,152 fragments of more than 1 km^2^, ten of which may support long-term viable populations (> 115 km^2^ cf. [43]). *Brachyteles hypoxanthus* occurs in three of these larger fragments (Itatiaia National Park, Rio Doce State Park and Serra do Brigadeiro State Park) [12,73], as well as some smaller, nearby fragments that together also amount to over 115 km^2^ of forest (Mata Escura Biological Reserve, Alto Cariri State Park and Caparaó National Park)[13], in addition to other isolated populations in smaller fragments (Fig 4A). Thus, surveys for remnant populations should focus on these seven large fragments and groups of fragments that add up to an area large enough to support viable, long-term (for 1,000 years) populations of muriquis, especially those that are in strictly protected areas where local extinction has not been documented (Fig 4A). To ensure population persistence, even in those larger fragments were the species remains, management at the population level might be needed to avoid genetic erosion [76]. Additionally, other, smaller, fragments may contain populations that are not viable over the long term (more than 50 years) and therefore need urgent management [52,77].

Only 20,611 km^2^ (12.9% of its historical distribution) of suitable forest habitat remains for *B*. *arachnoides*, but in contrast with *B*. *hypoxanthus*, large protected forest fragments are still found within its range [78]. Today, *B*. *arachnoides* populations are found in ten large fragments [13] (S2 Table) and SDM suggests another six locations with suitable habitat larger than 115 km^2^, with four of them in a continuum of restricted protected areas (Paranapiacaba Ecological Continuum and Serra do Mar State Park; Fig 4B). Unfortunately, occurrence in a large, continuous forested areas does not ensure conservation, because this species usually has a low population density [13,79,80] and still suffers pressure from poaching [12,13,81,82]. We identified another 1,801 fragments able to sustain populations, although *B*. *arachnoides* was found only in six [13,54,83], two in Paraná, which is its southern distribution limit. Therefore, additional and larger-scale surveys are needed, mainly where the occurrence of *B*. *arachnoides* is uncertain, but especially at its southern distribution limit, where little is known about their current conservation status.

## Conclusion

Potentially large and widely-distributed areas are suitable habitat for both species of muriqui, including areas where the species are known to be absent. By combining the distribution model and data from literature, we adjusted the historical range showing that in addition to environmental characteristics, two factors potentially shaped the limits of the historical distribution of muriqui species: physical (rivers and mountains as barriers to dispersal) and biotic (the two species seem mutually exclude one another). Based on this new perspective, the historical distribution proposed here was used as a starting point for estimating habitat loss and to identify areas for conservation concern where there is still no information on occurrence or absence (actual or historical). Thus, this study offers a way not just to find new muriqui populations but also to complement historical data which can bring new insights on the geographic distribution of these endangered species.

## Supporting Information

S1 Table Independent locations of historical and current occurrence of *Brachyteles hypoxanthus* used for modeling. (DOCX)

S2 Table Independent locations of historical and current occurrence of *Brachyteles arachnoides* used for modeling. (DOCX)

S3 Table Environmental variables used in the species distribution modeling of northern muriqui, *B*. *hypoxanthus*. (DOC)

S4 Table Environmental variables used in the species distribution modeling of southern muriqui, *B*. *arachnoides*. (DOC)
