# Supplementary material for: A Current Perspective on the Historical Geographic Distribution of the Endangered Muriquis (Brachyteles spp.): Implications for Conservation
Source: PLoS One. 2016 Mar 4;11(3):e0150906. doi: 10.1371/journal.pone.0150906 (PMC4778866; doi:10.1371/journal.pone.0150906)
Supplement: S1 Table — (DOCX) [file pone.0150906.s001.docx]

| **S1 Table. Independent locations of historical and current occurrence of *Brachyteles hypoxanthus* used for modeling.** | | | | | | |
| --- | --- | --- | --- | --- | --- | --- |
| ID | Location | Municipality | State | Longitude | Latitude | Reference |
| 1 | Congogi river | S. do Palhão - Gongongi | BA | -39.5000 | -14.3333 | MZUSP, tombo 3830 [1] |
| 2 | Riacho Duas Barras | Caatiba | BA | -40.233 | -15.0500 | [1] |
| 3 | Chapori, on the headwaters of Una river | Uma | BA | -39.6333 | -15.1167 | [1] |
| 4 | Serra Couro d'Anta | Itapetinga | BA | -39.8833 | -15.1833 | [1] |
| 5 | Córrego Mundo Novo | Pau Brasil | BA | -39.7167 | -15.3167 | [1] |
| 6 | Serra Pateirão | Encruzilhada | BA | -40.3000 | -15.4833 | [1] |
| 7 | Barreiras | Belmonte | BA | -39.3333 | -16.0500 | Museu Zoológico de Berlim, tombo 36458 [1] |
| 8 | Serra Gabiarra | Santa Cruz Cabrália | BA | -39.7500 | -16.0833 | [1] |
| 9 | Alto Cariri State Park | Salto da Divisa | MG | -39.9951 | -16.3149 | [2] |
| 10 | Mata Escura Federal Biological Reserve* | Jequitinhonha, Almenara | MG | -41.0117 | -16.3414 | [2] |
| 11 | RPPN Fazenda Duas Barras | Santa Maria do Salto | MG | -40.0543 | -16.4065 | [2] |
| 12 | Farinha Lavada e Água Limpa | Guaratinga e Jucuruçu | BA | -40.1667 | -16.9167 | [1] |
| 13 |  | Teófilo Otoni | MG | -41.5667 | -17.8333 | MZUSP, tombo 8582 [1] |
| 14 | Fazenda Córrego de Areia | Peçanha | MG | -42.7500 | -18.4333 | [3] |
| 15 | Faz. Pindorama, on Rio Doce margin | Linhares | ES | -40.0722 | -19.4222 | museu UFES [4] |
| 16 | Fazenda Jovem Arminda, Córrego dos Monos | Colatina | ES | -40.6255 | -19.5333 | museu UFES [4] |
| 17 | Rio Doce State Park* | Mariléia, Dionísio, Timótes | MG | -42.5667 | -19.6667 | [5] |
| 18 | Augusto Ruschi Biological Reserve | Santa Teresa | ES | -40.5500 | -19.9000 | [6] |
| 19 | RPPN Feliciano Miguel Abdalla | Caratinga | MG | -41.8167 | -19.7333 | [7] |
| 20 | Surroundings of Augusto Ruschi Biological Reserve | Santa Teresa | ES | -40.5333 | -19.8167 | [8] |
| 21 | Perobas | Ibiraçu (antiga Pau Gigante) | ES | -40.3750 | -19.8417 | museu UFES [4] |
| 22 | Jatibocas | Itarana | ES | -40.8333 | -19.8667 | museu UFES [4] |
| 23 | Sta. Lúcia Biological Station | Santa Teresa | ES | -40.6017 | -19.9361 | [9] |
| 24 | Private areas | Santa Maria do Jetibá | ES | -40.6833 | -20.0333 | [10] |
| 25 | R. Triunfo, near the headquarters | Santa Leopoldina | ES | -40.3200 | -20.0600 | museu UFES [4] |
| 26 | RPPN Mata do Sossego | Simonésia | MG | -42.0833 | -20.0667 | [5] |
| 27 | Faz. Rochedo | Rio Casca | MG | -42.8333 | -20.1333 | [1] |
| 28 | Brejetuba | Brejetuba | ES | -41.2758 | -20.1458 | museu UFES [4] |
| 29 | Rio das Pedras | Santa Leopoldina | ES | -40.6167 | -20.1500 | museu UFES [4] |
| 30 | Brejetuba | Afonso Cláudio | ES | -41.4000 | -20.2167 | [1] |
| 31 | Secondary Forest | Rio Casca | MG | -42.6506 | -20.2261 | ZUEC-MAM- Coleção de Mamíferos do Museu de Zoologia da UNICAMP [4] |
| 32 | Fazenda da Serra | Baixo Guandu | ES | -41.0833 | -20.2500 | museu UFES [4] |
| 33 | Duas Bocas Forest Reserve | Cariacica | ES | -40.5000 | -20.3000 | [9] |
| 34 | Fazenda Belon (4 Km do P. Est. Pedra Azul) | Domingos Martins | ES | -40.6592 | -20.3633 | museu UFES [4] |
| 35 | Pedra Azul | Domingos Martins e Alfredo Chaves | ES | -41.0117 | -20.4133 | [1] |
| 36 | Caparaó National Park* | Esperança Feliz Dores do Rio Preto, Divino de São Louranço, Ibitirama | MG / ES | -41.7500 | -20.4667 | [5] |
| 37 | Forno Grande | Castelo | ES | -41.1028 | -20.5028 | museu UFES [4] |
| 38 | - | Alfredo Chaves | ES | -40.7528 | -20.6361 | museu UFES [4] |
| 39 | Fazenda Muriquioca | Guarapari | ES | -40.5000 | -20.6667 | museu UFES [4] |
| 40 | Serra do Brigadeiro State Park* | Araponga, Fervedouro, Miradouro, Ervália, Sericita, Pedra Bonita, Muriaé, Divino | MG | -42.4833 | -20.7167 | [5] |
| 41 | Engenheiro Reeve (= Rive) | Alegre | ES | -41.4750 | -20.7750 | museu UFES [4] |
| 42 | Ibitipoca State Park | Lima Duarte | MG | -43.8833 | -21.7000 | [11] |
| 43 | Itatiaia National Park* | Resende, Itatiaia | RJ / MG | -44.6000 | -22.4333 | [12] |

*localities larger than 115 km^2^ (minimum size for supporting a viable population for the long term cf. [13]) with current occurrence of the species.

**REFERENCES**

1. Aguirre AC. O mono *Brachyteles arachnoides* (E. Geoffroy). Situação atual da espécie no Brasil. An Acad Bras Cienc. 1971;1–51.
2. Melo FR., Chiarello AG, Faria MB. Novos registros de muriqui-do-norte (*Brachyteles hypoxanthus*) no Vale do rio Jequitinhonha, Minas Gerais e Bahia. Neotrop Primates. 2004;12:139–42.
3. Hirsch A, Martins WP, Porfírio S. Rediscovery of *Brachyteles arachnoides hypoxanthus* at the Fazenda Córrego de Areia, Minas Gerais, Brazil. Neotrop Primates. 2002;10:119-122.
4. Database: specieslink [Internet]. Avaiable: http://splink.cria.org.br
5. Mendes SL, Santos RR, Carmo LP. Conservation of the northern muriqui in Santa Maria do Jequitibá, Espírito Santo. Neotrop Primates. 2005;13:31–6.
6. Talebi MG, Melo FR, Dias LG, Cunha AA, Mendes SL, Breves P, et al. Contextualização sobre *Brachyteles arachnoides* e *Brachyteles hypoxanthus*. In: Jerusalinsky L, Talebi MG, Melo FR, editors. Plano de Ação Nacional para a conservação dos muriquis. Brasília: ICMBio; 2011. p. 19–62.
7. Strier KB, Boubli JP. A History of Long-term Research and Conservation of Northern Muriquis (*Brachyteles hypoxanthus*) at the Estação Biológica de Caratinga / RPPN-FMA. Primate Conserv. 2006;20:53–63.
8. Vieira LA., Mendes SL. Presence of the muriqui (*Brachyteles hypoxanthus*) in a rural property in the vicinity of the Augusto Ruschi Biological Reserve, Santa Teresa, Espírito Santo. Neotrop Primates. 2005;13:37–9.
9. Mendes SL. Situação dos primatas em reservas florestais do estado do Espírito Santo. In: Rylands AB, Bernardes A, editors. A Primatologia no Brasil 3. Belo Horizonte: Fundação Biodiversitas; 1991. p. 347–56.
10. Mendes SL, Melo FR., Boubli JP, Dias LG, Stries KB, Pinto LPS, et al. Directives for the conservation of the northern muriqui, *Brachyteles hypoxanthus* (Primates, Atelidae). Neotrop Primates. 2005;13:7–18.
11. Fontes MAL, Oliveira-Filho AT, Galetti M. The muriqui in the Parque Estadual de Ibitipoca, Minas Gerais. Neotrop Primates. 1996;4:23–25.
12. Cunha AA, Grelle CEV, Boubli JP. Distribution, population size and conservation of the endemic muriquis (*Brachyteles* spp.) of the Brazilian Atlantic Forest. Oryx. 2009;43:254-257.
13. Brito D, Grelle CEV. Estimating minimum area of suitable habitat and viable population size for the northern muriqui (*Brachyteles hypoxanthus*). Biodivers Conserv. 2006;15:4197–4210.
