# Supplementary material for: A Current Perspective on the Historical Geographic Distribution of the Endangered Muriquis (Brachyteles spp.): Implications for Conservation
Source: PLoS One. 2016 Mar 4;11(3):e0150906. doi: 10.1371/journal.pone.0150906 (PMC4778866; doi:10.1371/journal.pone.0150906)
Supplement: S2 Table — (DOCX) [file pone.0150906.s002.docx]

| **S2 Table. Independent locations of historical and current occurrence of *Brachyteles arachnoides* used for modeling.** | | | | | | |
| --- | --- | --- | --- | --- | --- | --- |
| ID | Location | Municipality | State | Longitude | Latitude | Reference |
| 1 | Desengano State Park (Morumbeca)* | Santa Maria Madalena, São Fidélis, Campos dos Goytacazes | RJ | -41.8925 | -21.8917 | [1] |
| 2 |  | Boracéia | SP | -48.7833 | -22.1833 | MZUSP, tombo 7411 [2] |
| 3 | Rio Bonito | Silva Jardim, C. de Macacu, C. de Abreu e Friburgo | RJ | -42.4333 | -22.3667 | [2] |
| 4 | RPPN Guapiaçu/Rio Guapiaçu* | Cachoeiras de Macacu e Teresópolis | RJ | -42.7333 | -22.3833 | [3] |
| 5 | Serra dos Órgãos National Park* | Teresópolis, Guapimirim, Magé "Rio Soberbo" | RJ | -43.0167 | -22.4833 | [3] |
| 6 | Faz. do Subaio and Faz. do Carmo | Cachoeiras de Macacu | RJ | -42.8667 | -22.5000 | [2] |
| 7 | Fazenda Barreiro Rico | Anhembi | SP | -48.1000 | -22.6833 | [4] |
| 8 | RPPN Fazenda São Sebastião do Rio Grande | Pindamonhangaba | SP | -45.4667 | -22.7500 | [5] |
| 9 | Faz. do Veado e Serra da Bocaina | S. José do Barreiro e Bananal | SP | -44.8833 | -22.7667 | [2] |
| 10 | Bocaina National Park* | Paraty, Angra dos Reis, São José do Barreiro, Areias, Cunha, Ubatuba | RJ / SP | -44.2500 | -22.8333 | [6] |
| 11 | Fazenda Monte Verde and Fazenda Mandala | São Fracisco Xavier | SP | -45.9908 | -22.9011 | [7] |
| 12 | Alto Paraibuna | Ubatuba, S. Luís do Paraitinga e Paraty | SP/RJ | -44.9667 | -23.0000 | [2] |
| 13 | São Bartolomeu | Cerqueira César | SP | -49.2500 | -23.0167 | MZUSP [8] |
| 14 | APA Municipal de São Franciso Xavier | São José dos Campos | SP | -45.8667 | -23.2000 | [9] |
| 15 | APA do Cairuçu/ Pico do Cairuçu* | Paraty | RJ | -44.6333 | -23.3167 | [3] |
| 16 |  | Ipanema (hoje Varnhagem) – atual município de Iperó | SP | -47.7000 | -23.3333 | Museu Zoológico de Berlim, tombo 206 [2] |
| 17 |  | Ubatuba | SP | -45.0708 | -23.4339 | MZUSP [8] |
| 18 | Parque das Neblinas (RPPN Ecofuturo) | Bertioga | SP | -46.1500 | -23.7333 | [10] |
| 19 | Paranapiacaba District | Santo André | SP | -46.0594 | -23.8083 | MZUSP [8] |
| 20 | Serra do Mar State Park (núcleo cubatão)* | Cubatão | SP | -46.5167 | -23.9000 | [11] |
| 21 | Jurupará State Park | Ibiúna e Piedade | SP | -47.3000 | -23.9333 | [6] |
| 22 | - | Itararé | SP | -49.3500 | -24.1000 | MZUSP [8] |
| 23 | Fazenda Intervales | Serra da Paranapiacaba (Vale Carmo) | SP | -48.3833 | -24.1833 | [6] |
| 24 | Intervales State Park* | Guapiara, Ribeirão Grande, Sete Barras, Eldorado e Iporanga | SP | -48.1333 | -24.3000 | [12] |
| 25 | Juréia - Itatins | Peruíbe | SP | -47.0008 | -24.3131 | [8] |
| 26 | Fazenda Poço Grande | Juquiá | SP | -47.6333 | -24.3000 | MZUSP [8] |
| 27 | Alto Ribeira Tourist State Park* | Apiaí, Iporanga | SP | -48.6000 | -24.4500 | [10] |
| 28 | Carlos Botelho State Park* | São Miguel do Arcanjo, Capão Bonito, Sete Barras, Tapiraí | SP | -47.9667 | -24.1412 | [13] |
| 29 | Juréia Ecological Station - Itatins* | Iguape, Miracatu, Itariri, Peruíbe | SP | -47.2500 | -24.5000 | [6] |
| 30 | - | Iporanga | SP | -48.5928 | -24.5858 | MZUSP [8] |
| 31 | Fazenda Olho D’água | Doutor Ulysses | PR | -49.5044 | -24.6703 | B. Ingberman e N. Kaminski, pers. comm.[14] |
| 32 | Abapã | Castro | PR | -49.8611 | -24.8830 | [14] |
| 33 | Fazenda João Paulo II | Castro | PR | -49.6422 | -24.9689 | [15] |
| 34 | Fazenda Santana | Castro | PR | -49.9286 | -25.0197 | [14] |

*localities larger than 115 km^2^ (minimum size for supporting a viable population for the long term cf. [16]) with current occurrence of the species.

REFERENCES

1. Garcia VLA. Status of the Muriqui (*Brachyteles*) Populations Remainings in the State of Rio de Janeiro, Brazil: Projeto Muriqui-Rio. Neotrop Primates. 2005;13:73–8.
2. Aguirre AC. O mono *Brachyteles arachnoides* (E. Geoffroy). Situação atual da espécie no Brasil. An Acad Bras Cienc. 1971;1–51.
3. Cunha AA, Grelle CEV, Boubli JP. Distribution, population size and conservation of the endemic muriquis (*Brachyteles* spp.) of the Brazilian Atlantic Forest. Oryx. 2009;43:254-257.
4. Martins MM. Density of primates in four semi-deciduous forest fragments of São Paulo, Brazil. Biodivers Conserv. 2005;14:2321–9.
5. Oliveira MF, Manzatti L. New location for the muriqui (*Brachyteles arachnoides*) in the state of São Paulo, Brazil. Neotrop Primates. 1996;4:84–5.
6. Martuscelli P, Petroni LM, Olmos F. Fourteen new localities for the muriqui (Brachyteles arachnoides). Neotrop Primates. 1994;2:12–5.
7. Santos ASR. Encontros com os muriquis, *Brachyteles arachnoides* (E.Geoffroy,1806), de São Francisco Xavier, Serra da Mantiqueira, São Paulo, Brasil. [Internet]. 2011 [cited 2012 Jan 12]. Available: http://www.aultimaarcadenoe.com.br/wp-content/uploads/2011/06/Encontros-com-os-muriquis-SFX-AS.pdf
8. Database: specieslink [Internet]. Avaiable: http://splink.cria.org.br
9. Antonietto L, Mendes FDC. São Francisco Xavier: A new site for primatological research and conservation in the Brazilian Atlantic Forest. Neotrop Primates. 1994;2:3–4.
10. Talebi MG, Melo FR, Dias LG, Cunha AA, Mendes SL, Breves P, et al. Contextualização sobre *Brachyteles arachnoides* e *Brachyteles hypoxanthus*. In: Jerusalinsky L, Talebi MG, Melo FR, editors. Plano de Ação Nacional para a conservação dos muriquis. Brasília: ICMBio; 2011. p. 19–62.
11. Auricchio P, Silva M a. F. Nova ocorrencia de *Brachyteles arachnoides* no parque estadual da serra do mar, SP, Brazil. Neotrop Primates. 2000;8:30–1.
12. Talebi MG, Soares P. Conservation research on the southern muriqui (*Brachyteles arachnoides*) in São Paulo State, Brazil. Neotrop Primates. 2005;13:53–9.
13. Talebi MG. Field study of muriquis in the Carlos Botelho State Park, Brazil. Neotrop Primates. 1996;4(2):62–3.
14. Ingberman B. Fatores ecológicos de influência na distribuição geográfica de muriqui (*Brachyteles* Spix 1823) e bases para formulação de uma estratégia de conservação para o sul do Brasil. Ph.D. Thesis, Universidade Federal do Paraná. 2015.
15. Koehler AB, Pereira LCM, Nicola PA. New locality for the woolly spider monkey *Brachyteles arachnoides* (E. Geoffroy, 1806) in Parana state and the urgency of strategies for conservation. Estud Biol. 2002;24:25–9.
16. Brito D, Grelle CEV. Estimating minimum area of suitable habitat and viable population size for the northern muriqui (*Brachyteles hypoxanthus*). Biodivers Conserv. 2006;15:4197–4210.
