# Supplementary material for: A Current Perspective on the Historical Geographic Distribution of the Endangered Muriquis (Brachyteles spp.): Implications for Conservation
Source: PLoS One. 2016 Mar 4;11(3):e0150906. doi: 10.1371/journal.pone.0150906 (PMC4778866; doi:10.1371/journal.pone.0150906)
Supplement: S3 Table — (DOC) [file pone.0150906.s003.doc]

| **S3 Table. Environmental variables used in the species distribution modeling of northern muriqui, *B. hypoxanthus.*** | |
| --- | --- |
| Environmental variable | Percent contribution to the model |
| Temperature Seazonality | 56.1 |
| Isotermality | 13.7 |
| Altitude | 11.8 |
| Mean diurnal range | 6.5 |
| Precipitation of Driest Month | 6 |
| Annual Precipitation | 3.1 |
| Precipitation of Wettest Month | 2.8 |
