# Supplementary material for: A Current Perspective on the Historical Geographic Distribution of the Endangered Muriquis (Brachyteles spp.): Implications for Conservation
Source: PLoS One. 2016 Mar 4;11(3):e0150906. doi: 10.1371/journal.pone.0150906 (PMC4778866; doi:10.1371/journal.pone.0150906)
Supplement: S4 Table — (DOC) [file pone.0150906.s004.doc]

| **S4 Table. Environmental variables used in the species distribution modeling of southern muriqui, *B. arachnoides*.** | |
| --- | --- |
| Environmental variable | Percent contribution to the model |
| Precipitation of Driest Month | 17.7 |
| Isotermality | 17.3 |
| Mean Diurnal Range | 14.4 |
| Precipitation of Wettest Month | 13 |
| Annual Precipitation | 11.7 |
| Temperature Seazonality | 10.8 |
| Altitude | 10 |
| Annual Mean Temperature | 4.6 |
| Temperature Annual Range | 0.5 |
